# Neural Correlates of Mentalizing in Individuals With Clinical High Risk for Schizophrenia: ALE Meta-Analysis

**DOI:** 10.3389/fpsyt.2021.634015

**Published:** 2021-04-20

**Authors:** Ksenija Vucurovic, Stéphanie Caillies, Arthur Kaladjian

**Affiliations:** ^1^Laboratory Cognition, Santé, Société (C2S), Department of Psychology, University of Reims Champagne Ardenne, Reims, France; ^2^Centre Rémois de Psychothérapie et Neuromodulation, Reims, France; ^3^Pôle Universitaire de Psychiatrie, CHU de Reims, EPSM Marne, Reims, France

**Keywords:** theory of mind, schizophrenia, psychosis proneness, fMRI, social cognition

## Abstract

Psychotic disorder refers to a spectrum of disorders that have multiple etiologies, due to the complex interaction of biological and genetic vulnerability with familial and cultural factors. A clinical high risk (CHR) for schizophrenia is defined as the presence of brief, attenuated, or intermittent psychotic symptoms in non-schizophrenic individuals. The transition to schizophrenia appears significantly more frequent in this at-risk population than in the general population. Moreover, the ability to attribute mental states to others, known as mentalizing or theory of mind, and its neural correlates found in individuals with CHR are similar to those described in patients with schizophrenia. We have therefore explored neurofunctional correlates of mentalizing in individuals with CHR vs. healthy controls, in order to identify the differences in brain activation. A neural coordinate-based activation likelihood estimation meta-analysis of existing neuroimaging data revealed that three regions displayed decreased activation in individuals with CHR, compared with healthy controls: the right temporoparietal junction, the right middle temporal gyrus, and the left precuneus. These results, combined with those in the literature, further support the hypothesis that abnormal activation of posterior brain regions involved in mentalizing correlates with psychotic symptoms in help-seeking individuals.

## Introduction

Schizophrenia, a severe psychotic disorder, is among the leading causes of long-term disability worldwide (1). Therefore, better understanding of schizophrenia's emergence and disability correlates is one of the major challenges for modern psychiatric practice. Retrospective research has shown that individuals who develop schizophrenia display subclinical symptoms years before disease onset (2). Accordingly, the concept of clinical high risk (CHR) or ultra-high risk for psychotic disorders has emerged in the prospective literature (3, 4), and early interventions have been offered to help-seeking individuals in order to decrease their rate of transition to schizophrenia.

CHR state diagnosis is recognized as a prodromal period before schizophrenia onset and as such it is included in the latest version of Diagnostic and Statistical Manuel 5th version as “attenuated psychotic syndrome” (5). CHR is characterized by attenuated psychotic symptoms, brief limited intermittent psychotic symptoms, or basic symptoms (6). “Attenuated psychotic symptoms” refers to delusions, hallucinations, disorganized speech, or other psychotic symptoms present in an attenuated form. Reality testing is intact, but the symptoms are so frequent or severe that they cannot be ignored or discounted (7). Brief limited intermittent psychotic symptoms are frankly psychotic symptoms that do not last long enough for a diagnosis of schizophrenia to be made on the basis of current international criteria (8). Finally, basic symptoms are self-reported subtle, subclinical disturbances in stress tolerance and affective, cognitive, or perceptual drive, with full insight into their abnormal nature such that they motivate individuals to seek help (9, 10). Even though familial susceptibility to schizophrenia can be dissociated from CHR (11), it has been suggested that high genetic risk and functional decline are also diagnostic criteria for CHR (12).

It is thought that up to 20% of the general adult population have a psychotic-like experience (13). Nevertheless, individuals with CHR diagnostic criteria have a 16%−35% probability of developing a full-blown psychotic disorder within 2 years of CHR diagnosis (4, 14). Standardized validated instruments are used in clinical practice to identify individuals with CHR.

In schizophrenia, mentalizing is constantly impaired at both the behavioral and neurological levels (15, 16). Mentalizing (or theory of mind, ToM) is a social cognition process that confers on us the ability to attribute mental states to others and to further understand that these states may be different from ours (17). It enables individuals to make sense of social communication and interactions and to predict the social behavior of others (18). It has been suggested that the processes underlying mentalizing deficits are genetically influenced and may constitute an intermediate phenotype of schizophrenia (19–21). Consistent with this, both individuals with a high familial risk for schizophrenia (11) and those with CHR not only display poorer psychosocial functioning (22), but also perform more poorly on mentalizing tasks (23–27) and have abnormal brain activity compared with healthy controls (11, 28–34). For example, activation of the medial prefrontal cortex (mPFC) during a ToM task, specifically a false-belief task, has been found to be negatively correlated with social anhedonia and social functioning in CHR (35, 36), suggesting that the ToM processing impairment is related to relatively poor psychosocial functioning in this subclinical population.

The question now being asked is whether social cognition impairment is a marker of vulnerability to schizophrenia in individuals with CHR. Investigating the neural correlates of impaired mentalizing in CHR could make it possible to identify neuroimaging markers of psychotic disorders, which could then be added to the clinical criteria when screening at-risk individuals. This approach could help us identify useful targets for therapeutic interventions in psychosocial rehabilitation programs and, in turn, understand more fully the link between poor social cognitive functioning and the correlated brain network without a medication bias.

Even though several studies have already investigated the underlying ToM neural correlates in CHR, the small sample size of each study could lead to a lack of power. Reasoning that a meta-analysis of the existing neuroimaging data could help overcome this limitation, we carried out a systematic review of functional MRI studies of individuals with CHR, in order to examine which brain region activation in individual studies would keep robust association with TOM task resolution. We expected to find evidence of abnormal brain activation in core regions of the mentalizing brain previously shown as abnormally activated in schizophrenia during TOM task resolution, namely the bilateral temporoparietal junction (TPJ), left superior temporal sulcus, medial prefrontal cortex (mPFC), and precuneus.

## Methods and Materials

### Literature Search and Selection

We searched the following databases: MEDLINE, PsycINFO, Embase, and Current Contents. Relevant references from the retrieved papers were added to our database. Only whole-brain studies published in English until December 2020 were eligible for the review. We used the following keywords: *at-risk mental states, ARMS, clinical high risk, CHR, ultra-high risk, UHR, psychosis, mentalizing, theory-of mind, perspective-taking, fMRI, PET*, and *brain*. Paper selection is detailed in the PRISMA figure (Supplementary Material). Our procedure adhered to the 10 simple rules of Muller et al. (37) for neuroimaging meta-analysis. The present meta-analysis was registered in PROSPERO (no. CRD42019135862).

The inclusion criteria were (1) whole-brain neuroimaging (reported in the MNI or Talairach atlas) to compare individuals with CHR and healthy controls, (2) BOLD contrasts between the experimental condition (mentalizing) and the control condition, (3) mentalizing paradigm (featuring cartoons, video, or audio material) requiring participants to attribute mental states (emotions, intentions, beliefs) to others, and (4) diagnosis of CHR using validated clinical instruments and criteria (38).

Exclusion criteria were (1) CHR diagnosis based solely on familial susceptibility to schizophrenia, (2) brain damage or neurological disorder, (3) absence of a control group, and (4) mentalizing paradigm requiring either the attribution of a mental state to the self or emotion recognition, for although these processes are close to mentalizing, they do not fully overlap with the attribution of mental states to others (35, 39–42).

### Activation Likelihood Estimation Procedure

We submitted the neuroimaging data to a neural coordinate-based activation likelihood estimation (ALE) meta-analysis, using GingerALE version 2.3.6 (brainmap.org/ale) to run the ALE algorithm (43). We converted the coordinates from MNI space to the Talairach space using the *convert foci* option implemented in the GingerALE toolbox (44–46) and used the more conservative ALE method of Turkeltaub et al. (46) for the ALE calculation. We ran a cluster-level inference threshold correction algorithm (45), with *p* < 0.001 as the cluster-forming threshold and *p* < 0.05 for cluster-level inference. The cluster-level inference-corrected threshold set the minimum cluster volume such that only 5% of the simulated data's clusters exceeded this size. The minimum cluster size was 200 mm^3^. We performed the ALE analysis for all foci for ToM contrasts extracted from studies that compared individuals with CHR and healthy controls (HC).

## Results

**Figure 1:**
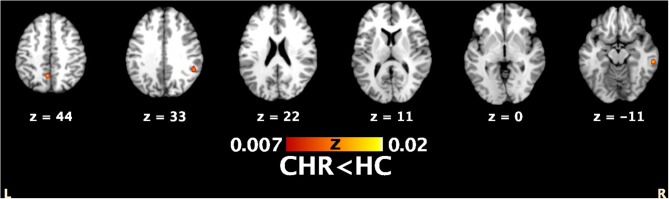
Functional clusters from comparison analysis between mentalizing networks showing significant neurofunctional convergence of decreased activation in individuals with CHR vs. healthy controls in following brain structures (from left to right): the left precuneus, the right parieto-temporal junction and right middle temporal gyrus. Mask dimensions = 80 × 96 × 70; number of within-brain voxels = 226,657; number of foci = 25; number of experiments = 7; maximum ALE score = 0.02; threshold method = cluster-level inference; thresholding value = 0.05; thresholding permutations = 1,000; cluster-forming value = 0.001. Figure created using Mango (http://ric.uthscsa.edu/mango).

The literature search yielded six studies (the research string is provided in Supplementary Figure 1), with seven experiments reporting 25 activation foci. Details about the studies we included, the paradigms they used, and the imaging characteristics are provided in Table 1. Two studies included individuals at familial high risk for schizophrenia (35, 47), but both studies focused on the clinical aspects of psychosis presented by the participants. Therefore, Marjoram et al. (47) compared individuals with high familial risk for schizophrenia with past or current attenuated psychotic symptoms with individuals with the comparable familial liability risk, but without those symptoms. Dodell-Feder et al. (35) included in their study the participants with both, familial liability to schizophrenia, and high scores on SIPS: Structured Interview for Prodromal Syndromes (Table 1). Therefore, the authors' consensus was that both studies meet typical CHR diagnostic criteria as defined by the field (48). A total of 91 individuals with CHR were compared with 110 HC. All the studies used fMRI. The ALE meta-analysis of the foci we extracted revealed decreased activation in the individuals with CHR, compared with HC, during mentalizing tasks in 1) a 960-mm^3^ cluster extending from the right inferior parietal lobule (Talairach: *x* = 46, *y* = −48, *z* = 26; ALE = 0.02; Brodmann area, BA 40) to the right supramarginal gyrus (Talairach: *x* = 54, *y* = −34, *z* = 34; ALE = 0.02; BA 40) and overlapping with the right TPJ (rTPJ), 2) a 560-mm^3^ cluster centered at *x* = 59, *y* = −30, *z* = −10 (Talairach) that covered the right middle temporal gyrus (MTG; BA 21), and 3) a 560-mm^3^ cluster that extended from (−10, −58, 38) to (0, −50, 46), centered at (−5, −54, 42), and overlapped with the precuneus. Detailed results are provided in Supplementary Table 1. All the regions of decreased activation are shown in Figure 1, and the link to the NIFTI file is: https://www.dropbox.com/s/ixpt8g5ykljmy9w/TOM%20HCvsHRP_ALE_C05_1k.nii?dl=0.

**Table 1:** Activation clusters.

**Cluster**	**Location (BA)**	**Talairach coordinates**			**ALE (× 10^−2^)**	**Cluster size (mm^3^)**
		**x**	**y**	**z**		
**HC>CHR**						
1	Right supramarginal gyrus (40)	50	−44	30	1,99	960
2	Right middle temporal Gyrus (21)	59	−30	−10	1,77	560
3	Left precuneus (7)	−5	−56	42	1,77	560

*BA, Brodmann Area; CHR, Clinical High Risk; HC, healthy controls; ALE, Activation Likelihood Estimation*.

*Centered cluster coordinates are reported*.

No significant cluster of increased activation was found in the individuals with CHR compared with HC.

## Discussion

We investigated the neural correlates of mentalizing in a population with CHR by conducting a meta-analysis of the neuroimaging literature. Even though only a small number of studies were eligible for quantitative meta-analysis, we obtained a significant result with good statistical robustness for three brain regions, which appeared to be insufficiently activated in comparison with HC: right TPJ, right MTG (BA 21), and left precuneus. All three regions are critically involved in the processing of social cognitive information in neurotypical development (49).

### Brain Function of Identified Areas

The right MTG (BA 21) was described as being involved in the processing of affective prosody in speech (50, 51), the auditory processing of complex sounds (52), visual memory function (53), and social cognition, particularly mentalizing during non-verbal tasks (54). The right TPJ is a highly integrative brain structure that enables high-order cognitive processing to take place. In social cognition, it is thought to be particularly involved in attention shift to unexpected stimuli and the attribution of intentions/beliefs to others (55). It has been suggested that the rTPJ can be divided into two functional domains: an anterior one involved in both ToM and attention and a posterior one that seems to be exclusively involved in ToM (55, 56). Disruption of the rTPJ impairs probabilistic belief updating (57). Finally, the precuneus is involved in a variety of cognitive processes, comprising the integration of environmental information and self-centered mental imagery strategies for its anterior portion (58), and episodic memory retrieval (58) and affective response to pain (59) for its posterior portion. The precuneus is reported to be involved in conscious information processing and closely connected to the mPFC, bilateral TPJ, and thalamus via the default mode network (60).

Our results are in line with volumetric studies of mentalizing in CHR that have found reduced gray-matter volume in the right superior temporal gyrus, left precuneus, left medial frontal gyrus, right middle frontal gyrus, bilateral parahippocampal/hippocampal regions and bilateral anterior cingulate, compared with HC (61). These authors further suggested that reduced gray-matter volume in the right inferior frontal gyrus and right superior temporal gyrus is predictive of the transition to psychotic disorder.

### Comparison With Genetic Risk for Schizophrenia

The literature indicates that individuals with familial high risk (FHR) for schizophrenia exhibit decreased mPFC activation and increased activation of the right MTG and posterior cingulate cortex during a mentalizing task (11). However, the results of fMRI studies comparing patients in acute psychosis and after recovery suggest that decreased mPFC activation is state-dependent (47, 62). In line with results observed in FHR, volumetric studies in schizophrenia have consistently described a positive correlation between ToM deficits and gray-matter reduction in the mPFC (63–68). In a large meta-analysis of ToM neural correlates, Schurz et al. (49) found that the mPFC is strongly activated in false-belief, strategic games, and trait judgment tasks and to a far lesser extent in social interactions, reading the mind in the eyes, and rational actions. The ventral mPFC is also described as being involved in processing explicit and implicit information about oneself and others in abstract and evaluative terms that enable individuals to understand complex psychological aspects of other people (69–72) or decouple mental states from their environment (73, 74). Barbey et al. (75) additionally claimed that the ventral mPFC specifically enables individuals to draw inferences about complex relations. It was therefore surprising not to find abnormal mPFC activation in our CHR meta-analysis. Even though some of the participants with CHR included in our meta-analysis had a genetic predisposition for schizophrenia, the major difference from relatives who were not affected by schizophrenia was the presence of attenuated psychotic symptoms, albeit at the subthreshold level. Consistently with this, Monhke et al. (11) demonstrated that activation of the right MTG and posterior cingulate cortex is correlated in FHR individuals with attenuated paranoid ideation. Abnormal mPFC activation during mentalizing tasks may therefore be an endophenotypic marker of schizophrenia related to genetic vulnerability (11), while abnormal activation of the temporoparietal cortex and precuneus may be related more to the psychopathology of psychotic disorders.

### Comparison With Schizophrenia

Three recent coordinate-based meta-analyses explored brain activation during mentalizing tasks resolution in schizophrenia compared to healthy controls [(76), 73, 74]. Both (76), and Kronbichler et al. (77), used Seed-Based Mapping methodology and concordantly found spread abnormalities in brain activation patterns in the patients group. Both groups of authors reported decreased activation of the mPFC, left orbitofrontal cortex, medial parieto-occipital cortex, right premotor areas, cingulate and lingual gyri [(76), 73]. Interestingly, in both meta-analyses, different activation of the bilateral parieto-temporal junction was described, with decreased activation of posterior ventral portion and increased activation in the posterior dorsal portion in schizophrenia patients [(76), 73], suggesting abnormal brain functioning of this area of the brain in patients. Our team led the comparative neural-correlates-based meta-analysis of mentalizing and empathy in schizophrenia patients using ALE methodology. We found decreased left TPJ activation during mentalizing tasks and decreased right ventrolateral PFC activation during emotion attribution tasks (78). Those literature data compared with the results of this meta-analysis in CHR, suggest abnormal activation in brain regions related to mentalizing in both schizophrenia and CHR, with diverging activation patterns in the right TPJ. Moreover, in patients with schizophrenia, the rTPJ is functionally connected to the hippocampus, fusiform gyrus, and MTG during mental state inference (79). Pauly et al. (80) found a positive correlation between positive symptoms and activation of the parahippocampus that led them to suggest a possible increase in emotion-related responses as the disease progresses. The opposite pattern of rTPJ activation in schizophrenia and CHR may therefore be related to more severe psychopathology in schizophrenia and more particularly to a cognitive mechanism of over-attribution of mental states or over-generation of hypotheses, described as *hyper-ToM* (81). Chambon et al. (82) postulated that the physiopathological mechanism, whereby undue weight is given to prior expectations in schizophrenia with positive symptoms, stems from the abnormal encoding of prediction error signals in dopamine-rich brain areas (83).

## Limitations and Conclusion

Our study had several limitations. As our meta-analysis was based on the available literature, it may have been affected by the potential publication bias against null results. Furthermore, the tasks used to assess neural correlates during mentalizing may have had limited ecological validity. Although very few experiments were included in our meta-analysis, we explored more than 20 foci, which is the threshold suggested by different authors for meta-analysis validity (37, 84).

In conclusion, our findings suggest that individuals with CHR may not have some fully functional specialized neural correlates for inferring the mental states of others. We found decreased activation of posterior brain areas related to mentalizing in these individuals that may be related to their increasing psychopathology. Longitudinal studies should address the neurological markers that predict the transition to schizophrenia in this population.

## Author Contributions

KV, SC, and AK contributed to the study conception and design. KV realized the systematic review of literature, done the first selection of relevant articles, data collection, statistical analysis, and drafted manuscript preparation. SC and AK done the final selection of included articles and supervised the research. All authors reviewed the results and approved the final version of the manuscript.

## Conflict of Interest

The authors declare that the research was conducted in the absence of any commercial or financial relationships that could be construed as a potential conflict of interest.
