# Supplementary material for: Neural Correlates of Mentalizing in Individuals With Clinical High Risk for Schizophrenia: ALE Meta-Analysis
Source: Front Psychiatry. 2021 Apr 20;12:634015. doi: 10.3389/fpsyt.2021.634015 (PMC8095711; doi:10.3389/fpsyt.2021.634015)
Supplement: Supplementary file 1 [file Data_Sheet_1.docx]

Supplementary Figure 1: PRISM figure illustrating research string.

Records identified through other sources

(N=3)

Records identified through database searching

(N=30)

Excluded articles after abstract screening

(N=15)

Records screened

(N=26)

Full-text articles excluded with reason (no whole brain data, connectivity studies…)

(N=5)

Full text articles assessed for eligibility

(N=11)

ELIGIBILITY

SCREENING

Duplicates removal

(N=26)

INCLUDED

Articles included to the meta-analysis

(N=6)

IDENTIFICATION

We searched the databases using all the following terms: theory of mind OR mentalizing OR false belief OR perspective taking AND fMRI OR PET OR brain AND CHR OR UHR OR ARMT OR psychosis proneness.
